# On the Preference of Local Bleeding to Vanæsection

**Published:** 1816-11

**Authors:** 


*For the London Medical and Physical Journal.*

### *On the Preference of local Bleeding to Venæsection;* by Chirurgicus.

AFTER having read Mr. Clark's eulogium, in your Journal for September, on that *potent* and *triumphant instrument* the *lancet*, (potent, God knows, and he might have added, often fatal instrument,) I was astonished at the conclusion of this case of Hernia humoralis. It seems that, from the 24th of June until the 3d of July, not less than *one hundred and eleven ounces of blood had been ordered to be drawn from the system, to affect a "part exterior to the body, and not essentially necessary to the functions of life."* It then occurred to him, after observing no good effect whatever on this "*exterior part,*" from the loss of *ninety-five* ounces of blood from the system, to call to his aid those *useful little animals, leeches,* to the number of ten: he proceeds to state, that, on the day after their use, his patient "*was not worse,*" nor *was the testicle much increased in size* (a circumstance which, by the bye, *did take place, after every previous bleeding from the system).* The leeches were repeated; and, "on the following day, he was agreeably surprised to find a gradual but decided improvement in all the symptoms," &c. His patient, he says, "*got rapidly well.*" Yet, Mr. Editor, he has not calculated on the remote effects on the constitution of his patient, by that wasteful plan with which he had commenced and continued the treatment of this very common disease. I could beg to ask Mr. Clark whether he can attribute this cure *solely* to those *large bleedings,* or whether to his *local* treatment. Had the leeches been used earlier, in numbers, and those repeatedly applied, I am confident his patient patient would have been cured by the loss of one-tenth of the blood which he so wantonly withdrew, in much less time, and without half the inconvenience which such a case produces.

If a residence " within the Lock Hospital," a situation where Mr. Clark must have seen this disease, has not already taught him this lesson, I beg that he will take it from *this*, his case, viz. that local evacuations are infinitely better than exhausting the system by such large bleedings from the system, a practice, which I regret to observe, making a most fearless progress, regardless of the ulterior effects on the constitution; a practice which all young *expectants*, and those with reversionary interest, will encourage: for these the lancet may well be styled *potent* and *triumphant*, for it sends many a one prematurely to the grave.

I am not a young man; I have lived to see the effects of the inconsiderate use of this " potent instrument," and I have great consolation in reflecting, that I have never used it *wantonly*. On this subject I may trouble you with my sentiments more in detail at no very great distance of time.

*London; Sept.* 24, 1816.

---

We are always thankful for communications from veterans in our art; and, contrary to our usual practice, have inserted the above, though anonymous, in answer to a gentleman who has honoured us with his name. In this case, an earlier attention to topical bleeding might have been desirable, but the texture of the part may have been preserved by the free bleeding from the arm, and, under convalescence, the system soon restores such a loss. We can boast years as well as Chirurgicus, and can even almost recollect when free bleeding was as common, and, we doubt not, as necessary, as at this time. Nor do we impute these variations in practice to mere fashion, but to an actual difference in the constitution of the air, which produces its effects on the human frame, as was accurately noticed by Sydenham. In our monthly reports, we have recommended topical bleeding as soon as the immediate danger of organic lesion has disappeared.—EDIT.

